# The Proximity between Hallucination and Delusion Dimensions: An Observational, Analytic, Cross-Sectional, Multicentre Study

**DOI:** 10.3389/fpsyg.2016.01642

**Published:** 2016-11-08

**Authors:** Diogo Telles-Correia, Ana L. Moreira, João Gama Marques, Sérgio Saraiva, Cátia A. Moreira, Filipa Antunes, Carolina Almeida, Nuno B. Rocha

**Affiliations:** ^1^Faculty of Medicine, University Clinic of Psychiatry and Medical Psychology, University of LisbonLisbon, Portugal; ^2^Schizophrenia Clinic, Hospital Júlio de Matos, Lisbon Psychiatric Hospital CenterLisbon, Portugal; ^3^School of Health Technologies, Polytechnic Institute of PortoPorto, Portugal

**Keywords:** Psyrats, hallucinations, psychopathology, psychosis, delusions

## Abstract

In psychiatric classifications, hallucinations (mainly auditory hallucinations) are one of the fundamental criteria for establishing a schizophrenia diagnosis or any of the related psychotic disorder's diagnoses. The conceptual proximity between delusions and hallucinations was maintained until the end of the XIX century, with several supporters during the XX century. Their limits were not yet definitely defined in terms of Descriptive Psychopathology, and much less so in terms of biochemical and anatomical models. In this article we aimed to analyse the dimensions of both hallucinations and delusions in a sample of patients with schizophrenia and schizoaffective disorder. We also intend to find the determinants of the main dimensions of hallucinations. One hundred patients with schizophrenia (80) or schizoaffective disorder (20), 64% males, mean age 39.75, from the outpatient and inpatient units of the Psychiatry Department of Hospital de Santa Maria and the Centro Hospitalar Psiquiátrico de Lisboa were assessed by means of the Psychotic Symptom Rating Scales (PSYRATS) and a structured interview. In this study we designed an empirical based model by means of bivariate Spearman's rank correlation coefficient, and multivariate statistics (linear regression and multiple multivariate linear regression), where the main dimensions of hallucinations are determined by the central dimensions of delusions.

## Introduction

Nowadays, hallucinations are classified according to their sensory modality: auditory, visual, tactile, cenesthetic, olfactory, gustatory, etc. (Telles-Correia et al., 2015).

Hallucinations may occur not only in functional psychotic states (schizophrenia, psychotic mania, psychotic depression), but also in organic situations or in cases of sensory deprivation (Telles-Correia et al., 2015).

In the current psychiatric classifications, hallucinations are one of the fundamental criteria for establishing a schizophrenia diagnosis or any of the related psychotic disorder's diagnoses (brief psychotic disorder, schizophreniform disorder, schizoaffective disorder). They may also be present in other psychiatric disorders, such as affective disorders (in depressive or manic episodes) (Telles-Correia et al., 2015).

Another symptom linked to the genesis of schizophrenia and other related psychotic disorders is delusion. Throughout the history of Psychiatry the conceptual proximity between delusions and hallucinations in the psychiatric patient was maintained until the end of the XIX century, with several supporters during the XX century such as Ey (1973), Ey et al. (1978). This means that their frontier was not yet definitely defined in terms of Descriptive Psychopathology, and much less so in terms of biochemical and anatomical models (Telles-Correia et al., 2015).

Contrary to former practice in which psychopathological description of symptoms was considered a priority, quantitative studies became a prime concern in the end of XX century. Nevertheless, in the last few years, with the re-emerging of Descriptive Psychopathology, several authors attempt to include again in their studies the more descriptive component of Psychopathology. Among these, the investigation of the several dimensions of delusions and hallucinations are highlighted (Chen and Berrios, 1996; Haddock et al., 1999; Telles-Correia et al., 2015).

Although many authors have suggested the conceptual proximity between hallucinations and delusions, we only found one preliminary empirical study which suggested a close relationship between the dimensions of the hallucinations and with the dimensions of delusion (Zanghellini Rückl et al., 2011).

In this article we aimed at analysing the dimensions of both hallucinations and delusions in an adult sample of patients with schizophrenia and schizoaffective disorder. We also intend to find the correlates of the main dimensions of hallucinations.

## Methods

### Participants

One hundred adult patients from both the outpatient and inpatient units of the Psychiatry Department of Hospital of Santa Maria and the Centro Hospitalar Psiquiátrico de Lisboa were included. This non-randomized but non-intentional (convenience) sample included all patients fulfilling the inclusion criteria and was collected by six medical doctors (three psychiatry residents and three psychiatrists) of both hospitals from July 2014 to July 2015. The same psychiatrists preformed the clinical assessment [all of them certified by the Portuguese Medical Association (Ordem dos Médicos Portugueses) and by the Ethics Committee of both centers]. They were assessed by means of a structured interview and PSYRATS. We first selected 118 patients, but 10 of them rejected to enter in the study and 8 to continue in the study. This group of dropouts didn't differ from the group investigated.

The inclusion criteria were the following: psychiatric patients with 18 years old or older, hospitalized or in ambulatory care; a schizophrenia or schizoaffective disorder diagnosis according to DSM-5; accepting to participate in the study and signing the informed consent. Exclusion criteria include: psychomotor agitation and compulsory treatment.

The study was approved by the Ethics Committee of both centers. Informed consent was obtained from all individual participants included in the study.

### Instruments

The Psychotic Symptom Rating Scales (Psyrats) were developed by Haddock, McCarron, Tarrier e Faragher (Haddock et al., 1999) to evaluate the dimensions of the two concepts: hallucinations (AVHs) and delusions (Haddock et al., 1999). The term dimension is used by the author of the scale meaning the characteristics of hallucinations and delusions (Haddock et al., 1999). They are composed by two subscales: one to evaluate the dimensions of hallucinations and another to evaluate the dimensions of delusions (Haddock et al., 1999). The Psyrats-hallucinations are composed by 11 items and each one is rated in an ordinal scale (from 0 to 5), varying from less to more severe. Each item corresponds to a dimension: frequency, duration, location (internal/external space), loudness, beliefs re-origin (*insight*), amount of negative content, degree of negative content, amount of distress, intensity of distress, disruption, and control. The subscale of delusions is composed by six items, each rated in the same way as the subscale of hallucinations. These six items correspond to the following dimensions: preoccupation, duration, conviction, amount of distress, intensity of distress, and disruption. The scale showed adequate psychometric characteristics, namely validity by criteria (converging) and inter-rater reliability (Close and Garety, 1998; Moritz and Larøi, 2008). Cronbach's alpha in our sample for the hallucinations' subscale was 0.84 and 0.85 for the delusions' subscale. This scale was validated by our study group for the Portuguese population. This validation was approved for publication and is going to be published briefly in “Actas Españolas de Psiquiatria.”

### Statistical analysis

We performed a spearman correlation coefficient bivariate analysis to evaluate the association of the dimensions of hallucinations within themselves and between them and the dimensions of delusions.

We also performed a stepwise linear regression to determine the determinants of the dimensions of hallucinations considered conceptually and clinically more important by the authors (as dependent variables). These were the ones that we considered more relevant from a theoretical point of view (according to the definitions that have been given throughout history of psychiatry): the location in space and the beliefs re-origin (that were the characteristics that more persistently have differentiated them from pseudohallucinations). From a clinical point of view it is the disruption that measures the global impact that the hallucinations have in the functioning of the patient. We used as independent variables the dimensions of hallucinations and of delusions that proved to be correlated to the former ones (in the bivariate analysis). We also included as independent variables sociodemographic factors (age; sex) and clinical elements (schizophrenia/schizoaffective disorder; with or without substance use; years since age of diagnosis and chlorpromazine equivalents) were included as independent variables. In each model of regression, problems related with multicollinearity were verified through the statistical test VIF (Variation Inflation Factor) that presented acceptable values in all cases (VIF <10).

We took in consideration in the interpretation of the results the Bonferroni Correction. Therefore the alpha accepted was 0.05/number of independent variables. Location in space (14 independent variables, *p* = 0.036) and the beliefs re-origin (20 independent variables, *p* = 0.025), disruption (19 independent variables, *p* = 0.026).

We used a multivariate multiple linear regression with AMEE (analysis of the model of structural equations) and the significance of indirect effects with the Sobel test (to check for the presence of mediator variables) to statistically find a theoretical model among the dimensions of hallucination and delusion.

The bivariate statistics and the regression models were exploratory but the AMME was directed to the findings of the former statistical analysis.

Since this is a cross sectional study no causal or explanatory relations are established.

Compliance with Ethical Standards:

Ethical approval: All procedures performed in studies involving human participants were in accordance with the ethical standards of the institutional and/or national research committee and with the 1964 Helsinki declaration and its later amendments or comparable ethical standards.
Informed consent: Informed consent was obtained from all individual participants included in the study.

## Results

### Clinical and sociodemographic characterization

The main clinical and sociodemographic variables are described in Table 1 Considering these variables, no statistically significant differences were found between the group of patients diagnosed with schizophrenia and the one diagnosed with schizoaffective disorder.

**Table 1:** **Sociodemographic and clinical variables variables**.

Sociodemographic and clinical variables	(N = 100)
Mean age	M = 39.751 SD = 12.403
Sex %	Female = 36%; Male = 64%
Diagnosis %	Schizophrenia: 80%; schizoaffective Dis.: 20%
Years since age of diagnosis (years/mean)	M = 11.721 SD = 10.452
Substance use	Cannabis = 45%, alcohol = 27%, cocaine = 17%, hallucinogens = 14%, heroin = 7.6.
Medical history	14% had obesity, 6% dyslipidemia, 5% high blood pressure, and 2% diabetes mellitus

### Psychopathological characterization

#### Global characterization of hallucinations and delusions

Concerning psychopathological characterization of the 100 patients evaluated; only 24% did not show any type of hallucination at the moment of observation. In 67% of all cases AVHs were present, and in 9% other type of hallucinations were present (in the absence of AVHs). All patients with AVHs had also delusions.

Auditory verbal hallucinations were present in the first person in 37% of the cases, in the second person in 58%, in the third person in 52%, and in 43% of the cases only isolated words were present.

Only 15% of all patients was non-delusional at the time of evaluation. In 51% a delusion with a persecutory theme was present (usually associated to other themes, such as mystical/religious, erotomaniac, ruin, grandiose, poisoning, reference), and in 31% a mystical/religious theme was present (most cases were associated to a persecutory, grandiose or erotomaniac theme).

Considering these variables, no statistically significant differences were found between the group of patients diagnosed with schizophrenia and those diagnosed with schizoaffective disorder.

#### Characterization of the current dimensions of hallucinations and delusion according to PSYRATS

Characterization of the Current Dimensions of Hallucinations and Delusion According to PSYRATS Are Described in Tables 2, 3.

**Table 2:** **Characterization of the current dimensions of hallucinations according to PSYRATS**.

**Psyrats hallucinations**	**Mean/Std. Dev**
Psyrats 1	1.5/1.47
Psyrats 2	1.65/1.52
Psyrats 3	1.98/1.75
Psyrats 4	1.46/1.27
Psyrats 5	2.11/1.73
Psyrats 6	1.71/1.58
Psyrats 7	1.73/1.71
Psyrats 8	1.77/1.72
Psyrats 9	1.68/1.60
Psyrats 10	2.07/1.67
Psyrats 11	2.15/1.70

**Table 3:** **Characterization of the current dimensions of delusions according to PSYRATS**.

**Psyrats delusions**	**Mean/Std. Dev**
Psyrats 1	1.93/1.46
Psyrats 2	2.26/1.45
Psyrats 3	2.75/1.62
Psyrats 4	1.91/1.62
Psyrats 5	1.79/1.51
Psyrats 6	2.57/1.51

#### Determinants of beliefs re-origin, location, and disruption of the hallucination

To find statistical models of correlation and with an adjustment of confounding variables we used a linear regression analysis (using the *stepwise* method). As independent variables we used the dimensions of hallucinations and delusions for which we obtained a statistically significant correlation in the bivariate analysis with the dependent variable (Tables 4, 5). We also included as independent variables sociodemographic (age, gender) and clinical factors (schizophrenia/schizoaffective disorder; with or without psychiatric history, with or without substance use, years since age of diagnosis and chlorpromazine equivalents).

**Table 4:** **Correlation between hallucinations' dimensions**.

**Psyrats Hall. (Spearman coef./p)**	**1**	**2**	**3**	**4**	**5**	**6**	**7**	**8**	**9**	**10**	**11**
1	_____		X	X		X					
		0.715			0.459		0.403	0.403	0.293	0.343	0.317
		0.000			0.000		0.000	0.000	0.012	0.003	0.007
2		_____									
	0.715		0.316	0.326	0.384	0.293	0.396	0.469	0.432	0.395	0.469
	0.000		0.007	0.005	0.001	0.012	0.001	0.000	0.000	0.001	0.000
3	X		_____	X							
		0.316			0.397	0.267	0.243	0.247	0.281	0.250	0.327
		0.007			0.001	0.023	0.039	0.036	0.017	0.034	0.005
4	X		X	_____				X		X	
		0.326			0.249	0.466	0.286		0.318		0.412
		0.005			0.035	0.000	0.015		0.006		0.000
5					_____	X		X	X		
	0.459	0.384	0.397	0.249			0.298			0.401	0.341
	0.000	0.001	0.001	0.035			0.011			0.000	0.003
6	X				X	___					
		0.293	0.267	0.466			0.731	0.642	0.649	0.352	0.274
		0.012	0.023	0.000			0.000	0.000	0.000	0.002	0.012
7							___				
	0.429	0.396	0.243	0.286	0.298	0.731		0.664	0.681	0.448	0.354
	0.000	0.001	0.039	0.015	0.011	0.000		0.000	0.000	0.000	0.002
8				X	X			___			
	0.403	0.469	0.247			0.642	0.664		0.862	0.544	0.522
	0.000	0.000	0.017			0.000	,000		0.000	0.000	0.000
9					X				___		
	0.293	0.432	0.281	0.318		0.649	0.681	0.862		0.459	0.518
	0.000	0.000	0.017	0.006		0.000	0.000	0.000		0.000	0.000
10				X						___	
	0.343	0.395	0.250		0.401	0.352	0.448	0.544	0.459		0.435
	0.003	0.001	0.034		0.000	0.002	0.000	0.000	0.000		0.000
11											___
	0.317	0.469	0.327	0.412	0.341	0.274	0.354	0.522	0.518	0.435	
	0.007	0.000	0.005	0.000	0.003	0.02	0.002	0.000	0.000	0.000	

*Legend: Psyrats Hall., Psyrats Hallucinations subscale*.

**Table 5:** **Correlation bettween hallucinations' dimensions and delusions' dimensions**.

	**1PD**	**2PD**	**3PD**	**4PD**	**5PD**	**PD**
1PH	0.253				0.254	
	*p* = 0.032				*p* = 0.031	
2PH.	0.33			0.393	0.370	
	*p* = 0.005			*p* = 0.001	*p* = 0.001	
3PH	0.343		0.233	0.368	0.319	
	*p* = 0.003		*p* = 0.48	*p* = 0.001	0.006	
4PH	0.57					
	*p* = 0.029					
5PH	0.508	0.339	0.409	0.310	0.294	0.421
	*p* = 0.000	*p* = 0.004	*p* = 0.000	*p* = 0.008	*p* = 0.012	*p* = 0.000
6PH				0.330	0.379	
				*p* = 0.005	*p* = 0.001	
7PH				0.328	0.429	
				*p* = 0.000	*p* = 0.000	
8PH				0.587	0.597	0.290
				*p* = 0.000	0.000	*p* = 0.13
9PH				0.543	0.609	
				*p* = 0.000	*p* = 0.000	
10PH	0.405	0.397		0.349	0.388	0.617
	*p* = 0.000	*p* = 0.001		0.003	*p* = 0.001	*p* = 0.000
11PH				0.461	0.377	0.261
				*p* = 0.000	*p* = 0.001	*p* = 0.027

*Legend: PH, Psyrats Hallucinations subscale; PD, Psyrats Delusions subscale*.

For the dimension “beliefs re-origin” we obtained a model of regression with a *R*^2^ of 50, which means that it explained 50% of the variance of this variable. In this model, some variables of the hallucination scale were present, but the most important determinant was the variable “Conviction” (*p* = 0.000). Other factors that predicted this dimension were the frequency of the hallucinations (*p* = 0.003), the control of the hallucinations (*p* = 0.014) and the amount of negative content (*p* = 0.036; Table 6).

**Table 6:** **Determinants of the main dimensions of the hallucination—linear regression**.

		***R*^2^**
Beliefs re-origin	Conviction — *p* = 0.000; *B* = 0.424	0.500
	Frequency — *p* = 0.003; *B* = 0.307; Control — *p* = 0.014; *B* = 0.242; cont neg — *p* = 0.036; *B* = 0.221	
Location	Beliefs re-origin — *p* = 0.000; *B* = 0.390	0.616
	Amount of negative content — *p* = 0.004; *B* = 0.282; Control — *p* = 0.034; *B* = 0.218	
Disruption	Beliefs re-origin — *p* = 0.000; *B* = 0.495	0.786
	Amount of distress caused by the hallucination — *p* = 0.000; *B* = 0.472; Years since age of diagnosis — *p* = 0.023; *B* = 0.017	

Regarding the dimension “Location of the hallucination” we obtained a regression model with an *R*^2^ of 0.616, which means that it explains 61.6% of the variance of this variable. In this model the most important determinant was the beliefs re-origin (*p* = 0.000), other determinant factors were the control (*p* = 0.034) and the amount of negative content (*p* = 0.004) (Table 1). According to Bonferroni Correction we could not accept this last determinant (*p* = 0.034, >0.025).

Considering the dimension disruption, we obtained a regression model with an *R*^2^ of 0.786, which means that it explains 78.6% of the variance of this variable. The main determinant factor was again the beliefs re-origin (*p* = 0.000, *B* = 0.495). Other determinant factors were the amount of distress caused by the hallucination (*p* = 0.000) and the years since age of diagnosis (*p* = 0.023; Table 1).

Considering the results of the previous regression models, an integrated model was proposed between the main dimensions of the hallucination and the conviction of the delusion. We used a multiple multivariate linear regression AMEE, with an estimation of the parameters by the method of maximum likelihood that was implemented in the AMOS v 16 SPSS software.

**Figure 1:**
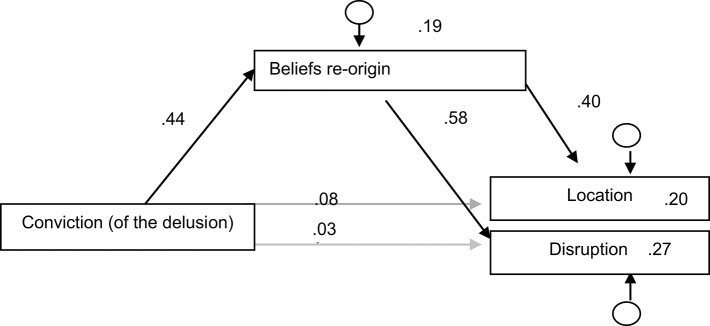
**Output of the multiple multivariate linear regression with AMEE**.

In this model, all trajectories were confirmed as statistically significant, except the direct connection between the conviction and the disruption. The model explains 20% of the variance of the location, 27% of the variance of the disruption, and 19% of the variance of the beliefs re-origin (Figure 1).

We infer from this model that the beliefs re-origin could then be an intermediary variable between the conviction and the location and disruption of the hallucination.

The significance of the indirect effect of the conviction (of the delusion) over the location mediated by the beliefs re-origin is statistically significant (*p* = 0.008). The significance of the indirect effects was evaluated with the Sobel test.

The significance of the indirect effect of the conviction over the disruption intermediated by the beliefs re-origin is statistically significant (*p* = 0.001). The significance of the indirect effects was evaluated with the Sobel test. Thus, we confirm this intermediary.

**Figure 2:**
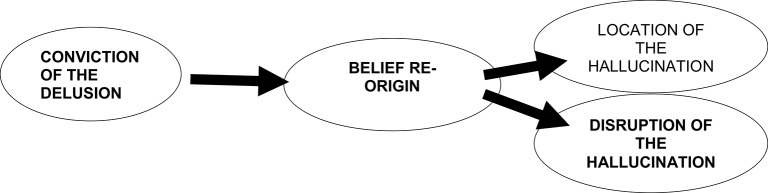
**Model of the determinants of the main dimensions of the auditory verbal hallucinations in patients with schizophrenia and schizoaffective disorder**.

As we have referred above, these conclusions do not allow us to infer a causal or explanatory model. Although the search for the significance of indirect effects with the Sobel test (to check for the presence of mediator variables) does allow us to infer about the direction of the statistical relations showed in the Figure 1. Based on these data we have drawn a Model of the determinants of the main dimensions of the auditory verbal hallucinations (Figure 2).

## Discussion and conclusion

Based on our findings in the explorative multiple regression models we considered a hypothesis of interaction among the main dimensions of the hallucination and the delusion. Within this framework a model was proposed recurring to a multiple multivariate linear regression with AMEE, and the significance of the indirect effects was evaluated with the Sobel test, to know the presence of intermediary variables, which allowed us to infer about the direction of the statistical relations (Figure 1).

Through this correlational model there seems to be a strategic determination of the conviction (of the delusion) about the belief re-origin that determines, in its turn, the most important dimensions of the hallucination.

The conclusions of our study empirically corroborate the conceptual references that have always approximated both concepts: hallucinations and delusions. Many authors argued about the validity and the boundaries of the concept hallucination. Since Esquirol (Esquirol, 1845; Telles-Correia et al., 2015), the author that integrated the term hallucinations in psychiatry, that the boundaries between delusions and hallucinations are not so clear. In the first half of the XX century also other authors such as Henry Ey did shared the same point of view (Ey, 1973).

Although many conceptual studies invoke the proximity between the concepts of hallucination and delusion, few are those that in an empiric way try to establish this relationship. There is a recent tendency to investigate the causes of hallucination in an independent way as if they were separate entities of the rest of the psychotic experience. If, in the case of a localized biological cause being present, the search for a primary anatomical focus of the hallucination may be linear, in psychiatric patients with a transversal psychotic experience (such as in schizophrenia) that affects the whole mental structure of the individual, it is difficult to find specific models of the hallucination. Thus, it is possible that there is here a profound proximity between concepts such as delusion and hallucination, from which content of the connection remains to be explained. Can they be interpreted as emerging simultaneously? May the delusion be the basis of the psychotic experience and the hallucination a type or a consequence of the delusion (as classic authors stated)? Or are hallucination and delusion consequences of a previous cause to both of these psychopathological experiences as, for instance, the disturbances in the boundaries of the self? Could both AVHs and delusions be result of other shared cause such as loss of the deictic anchoring? (using Crow's expression; Crow, 1997).

These conclusions can be important not only to a deep reflection about the validity of these concepts in psychopathology (Zachar, 2012) but also to bring some insights about the key phenotypes that should be included in the neuroimaging and neurobiological studies. Should the biological correlates of delusions and hallucinations be investigated separately?

According to several authors the pursuit of the biological correlates of psychiatric diseases can only be based on symptoms and not in disorders (Jablensky, 2010; Berrios, 2011). Therefore it is necessary that the psychopathological investigation begins with a profound conceptual analysis that illuminates the paths that should be followed and that prevent an investment in fruitless trajectories. Only after and with this orientation, empirical studies should be drawn with a psychometric and statistical basis (as is the case of this one) or anatomobiological basis (where anatomical and biological basis are searched).

We only found a study (Zanghellini Rückl et al., 2011) in the literature in which, in an empirical way, the dimensions of the hallucinations and the delusions were correlated.

In our study we pursued the evaluation not only of the degree of statistical relationship between the dimensions of the hallucinations, but also tried to establish a directionality of this relationship with the specific statistical methods such the Sobel test, to know the presence of intermediary variables, In this study, we achieved the establishment of deterministic models for the different dimensions of the hallucinations, and demonstrated the central role of the conviction of the delusion.

Among the main limitations of this article we highlight the reduced dimension of the sample (*n* = 100) and the fact that the design of the study (namely being a cross sectional study) did not allow us to draw a causal or explanatory model but only a correlational one.

## Author contributions

All authors listed, have made substantial, direct and intellectual contribution to the work, and approved it for publication.

### Conflict of interest statement

The authors declare that the research was conducted in the absence of any commercial or financial relationships that could be construed as a potential conflict of interest. The reviewer AS declared a shared affiliation, though no other collaboration, with several of the authors DT, AM, JG, SS, FA, CA to the handling Editor, who ensured that the process nevertheless met the standards of a fair and objective review.
